# Cardio-Protective Role of a Gut Hormone Obestatin: A Narrative Review

**DOI:** 10.7759/cureus.37972

**Published:** 2023-04-22

**Authors:** Rajal R Bora, Roshan Prasad, Mahalaqua Nazli Khatib

**Affiliations:** 1 Physiology, Jawaharlal Nehru Medical College, Datta Meghe Institute of Higher Education and Research, Wardha, IND; 2 Medicine and Surgery, Jawaharlal Nehru Medical College, Datta Meghe Institute of Higher Education and Research, Wardha, IND; 3 Epidemiology and Public Health, Jawaharlal Nehru Medical College, Datta Meghe Institute of Higher Education and Research, Wardha, IND

**Keywords:** obestatin, cardio-protection, ischemia reperfusion injury, ghrelin obestatin ratio, blood pressure

## Abstract

Obestatin is a gut hormone composed of 23 amino acids that play a role in protecting the heart. It is synthesized from the same preproghrelin gut hormone gene as another gut hormone. The function and receptor of obestatin remain controversial, despite being present in various organs such as the liver, heart, mammary gland, pancreas, and more. The activity of obestatin is opposite to that of ghrelin, another hormone. The GPR-39 receptor is used by obestatin to exert its effects. Obestatin's cardioprotective role can be attributed to its ability to affect various factors, including adipose tissue, blood pressure regulation, heart, ischemia-reperfusion injury, endothelial cells, and diabetes. Because these factors are related to the cardiovascular system, modifying them via obestatin can provide cardioprotection. Furthermore, ghrelin, its antagonist hormone, regulates cardiovascular health. Diabetes mellitus, hypertension, and ischemia-reperfusion injury can all alter ghrelin/obestatin levels. Obestatin has also been shown to impact other organs, reducing weight and appetite, inhibiting food intake, and increasing adipogenesis. Obestatin has a brief half-life and is quickly degraded by proteases in the blood, liver, and kidneys after entering circulation. This article offers insights into the cardiac function of obestatin.

## Introduction and background

Obestatin is a 23 amino acid amidated peptide encoded by the same gene as ghrelin [1]. It is produced in the gastrointestinal tract, particularly in the stomach. Still, its presence is marked in its production site and the spleen, mammary gland, breastmilk, and plasma [2]. It is a part of the complex gut-brain network which signals the brain regarding satiety and hunger. Besides its role in hunger and satiety, it also improves memory, regulates sleep and cell proliferation, affects pancreatic juice secretion, and inhibits glucose-induced insulin secretion. It is an anorexigenic peptide and ligand for GPR-39, an orphan G-protein-coupled receptor (GPCR). Obestatin opposes growth hormone secretion and ghrelin-induced food intake [3]. It is one of the gastrointestinal hormones produced by post-translational processing of a precursor preproghrelin. Besides its cardioprotective action, it has several other activities in the body. In the heart, it increases vasopressin release, produces vasodilation induced by nitric oxide, and has cardioprotective action [4]. Whereas in the brain, it decreases growth hormone secretion, reduces the severity of epileptic seizures, decreases the damage to dopaminergic neurons, and increases memory retention. The action of obestatin on the pancreas is to increase insulin secretion, increase beta cell function, and improves healing from acute pancreatitis. In fat cells, it acts opposite to ghrelin by increasing lipolysis and increasing GLUT4 translocation. In the gastrointestinal tract, it decreases motility, decreases appetite, increases water intake, and increases the healing of gastric ulcers. In muscles, it increases myogenic differentiation, muscle regeneration, micro vascularization, and mitochondrial biogenesis. It also activates pathways like phosphoinositide 3-kinases (PI3K) and EPK1/2 in the beta cells of the pancreas, which are antiapoptotic signaling pathways. The kinases act against myocardial injury and, thus, have a cardioprotective role [5]. In addition to its action, it also affects feeding, weight gain, gastrointestinal motility, memory, anxiety, and sleep. In addition, it stimulates the proliferation of human retinal pigment. In rats, it is found in the gastrointestinal tract within A-like cells, in the oxyntic glands of gastric mucosa, and in Leydig cells of the testis, where it is present along with its precursor peptide [6].

**Figure 1:**
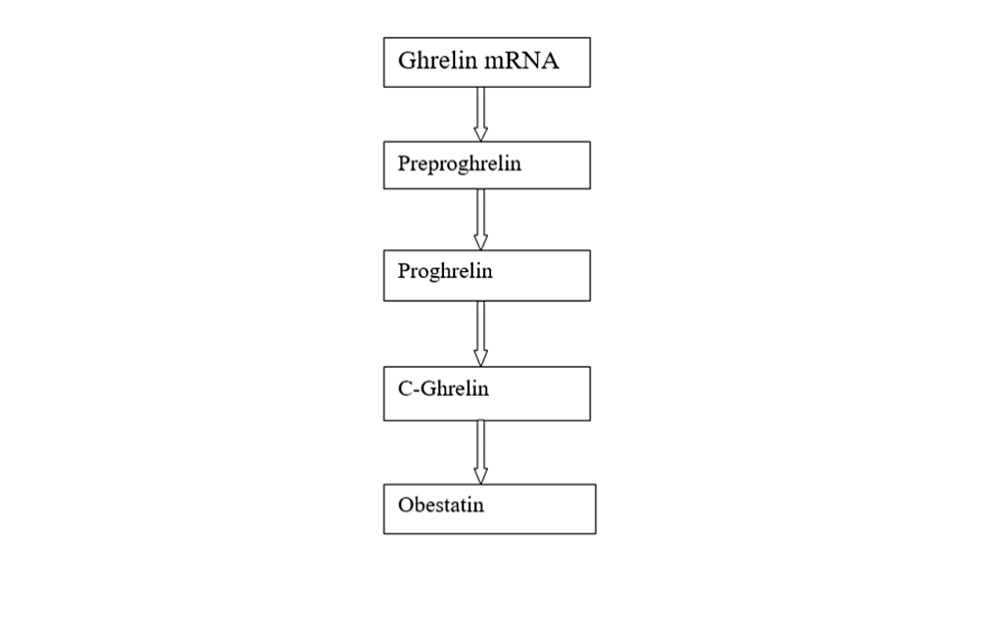
Flowchart showing the synthesis of obestatin The author has recreated from the source [8].

A recent study supports the relationship between obestatin and blood pressure. In patients with pulmonary arterial hypertension, obestatin levels were raised while the ghrelin/obestatin ratio was decreased. In hypertensive rats, the fasting obestatin level was increased, but the ghrelin/obestatin ratio was also elevated. In addition to its physiological action, it is useful in some gastrointestinal diseases. In rats, it prevents ulcerative colitis by inhibiting lipid peroxidation and reducing inflammation mediated by TH-1. In the pancreas, it enhances the generation of islet clusters with increased insulin gene expression. It protects against acute pancreatitis in rats by improving blood supply to the pancreas and reducing inflammation. It also induces pancreatic repair and regeneration. In adipocytes, it inhibits apoptosis and increases survival by stimulating pathways. Glucose uptake is also enhanced by human subcutaneous adipocytes with an increased translocation of GLUT-4 to the plasma membrane [7,8]. Figure 1 shows the synthesis of obestatin in the form of a flowchart.

## Review

Methodology

This review article underwent a rigorous scientific literature search using multiple electronic databases, namely PubMed, Embase, and Google Scholar. The search strategy involved a combination of keywords and medical subject headings (MeSH) terms, including "obestatin," "cardio-protection," "ischemia-reperfusion injury," "ghrelin obestatin ratio," and "blood pressure." The inclusion criteria for the selected articles were original research articles, review articles, meta-analyses, articles related to endometrial receptivity, implantation, and infertility, as well as articles focusing on the menstrual cycle, hormones, molecular mechanisms, biomarkers, and assisted reproductive techniques. These articles were published in peer-reviewed journals and written in the English language within the period from 2000 to 2022. On the other hand, exclusion criteria were defined as articles not related to the review topic, articles published in non-peer-reviewed journals, articles published in languages other than English, duplicate articles, and articles published before 2000 and after 2022. The rigorous search methodology and inclusion/exclusion criteria ensured that the review article was based on high-quality scientific literature that met the specific requirements of the research question.

Role of obestatin

Obestatin plays a significant role in the cardiovascular system in physiological and pathological conditions. High levels of obestatin were found in overweight patients with heart diseases compared to healthy people. Still, the serum level decreased in people with type 2 diabetes mellitus and obesity. Ghrelin, another gut hormone, has opposite actions to that of obestatin. For instance, obestatin decreases gastric motility and emptying, while ghrelin increases appetite, food intake, gastric secretions, emptying, and motility. Ghrelin is also associated with the cardioprotective role. The altered ratio of ghrelin/obestatin was seen in patients with chronic heart failure with cachexia [9].

Obestatin improves the function of the myocardium and decreases cell death and prevents apoptosis of cardiac cells, that is, cardiomyocytes, after ischemia/reperfusion. These procedures are performed by obestatin receptors present in cardiac cells. There is also the activation of signaling pathways in reperfusion injury salvage kinase (RISK), which includes PI3K and protein kinase C (ERK). In patients with diabetes mellitus, hyperglycemia is an important element that leads to cardiac dysfunction. It leads to the production of reactive oxygen species, which leads to this cardiac fibrosis and contractile dysfunction. Here, the protective role is being played by obestatin against this dysfunction. Obestatin acts by reducing contractility and decreasing the response of beta-adrenergic receptors. It restores oxidative balance and promotes modulation of AMPK and prosurvival kinase-like AKT, EPK1/2, and glycogen synthase kinase [10,11]. Table 1 shows the action of obestatin at different sites.

**Table 1:** Actions of obestatin at different sites The author has recreated from the source [11].

Site	Action
Heart	Decreases cardio-myocyte apoptosis and increases cardio-protection
Central nervous system	Decreases food intake and decreases thirst
Gastro-intestinal system	Decreases motility and gastric emptying
Muscles	Increases myogenesis and muscle regeneration
Pancreas	Decreases apoptosis and increases insulin secretion

GPR-39 receptor of obestatin

The receptor of obestatin is GPR-39. It is a GPCR and also the receptor for ghrelin and motilin [12]. It is an orphan member of the ghrelin receptor family and is recently recognized as a receptor for obestatin. In the polymerase chain reaction, the expression of GPR-39 was seen in peripheral organs like the duodenum, kidney, and heart [13]. The growth hormone secretagogue has two members: GPR-39 and GPR-38. It is also expressed in the central nervous system along with peripheral organs. It is signaled with constitutive activity via Gq and G12/13 signaling pathways. It is cloned from the human stomach cDNA library. GPR-39 stimulates cAMP production when exposed to zinc, but no reproducible activity is seen via the Ga signaling pathway [14].

GPCRs have seven transmembrane canonical architectures, which allow them to sense various stimuli, be they sensory (vision and taste) or those synthesized by the body that balance physiological response [12]. Zinc, a cofactor and messenger for various cellular functions, acts as a modulator of GPR-39. It is the dual sensor of two eicosanoids with opposite actions on microvascular tone, and its activities are vasodilation and vasoconstriction. It is a 230 kilobase molecule that is present on 2q21-q22. The locus of GPR-39 encodes two exons that synthesize two variants. The first variant includes both exons and synthesizes full-length receptors with seven transmembrane architectures related to GPCRs. It is responsive to many natural and synthetic ligands via downstream signaling pathways. The second isoform encloses only the first exon of the locus and encodes proteins containing the first five trans-membrane domains [15].

Obestatin in diabetes mellitus

There is a high risk of developing obesity-related type 2 diabetes. It was a major cause of death for about 5 million adults in 2015, with major cardiovascular system complications like stroke, neuropathy, and atherosclerosis. A large number of deaths are occurring despite the established therapies. Newer drugs have been developed targeting glucagon-like peptide (GCP-1) receptors. It is known to improve the metabolic profile and decrease the cardiovascular risk in diabetic individuals [16]. Type 2 diabetes mellitus increases the risk of atherosclerosis; therefore, controlling it is important in improving cardiovascular health. In recent years there has been increased cardiovascular morbidity, which is a significant cardiometabolic risk and has an essential role in the pathophysiology of arteriosclerosis, diabetes, and hormones like ghrelin and obestatin. Obestatin is correlated with intima-media thickness, a biomarker of atherosclerosis, suggesting that obestatin plays an essential role in inhibiting carotid atherosclerosis at an early stage. It also improves myocardial function. It is related to insulin resistance and metabolic dysfunction and inhibits food intake and body weight gain [17].

Patients with type 2 diabetes mellitus have lower levels of obestatin compared to the group of normal glucose tolerance and impaired glucose tolerance population. At the same time, the obestatin level in the impaired glucose tolerance population is lower than the average glucose tolerance population. Obestatin is regarded as a nutritional marker reflecting insulin resistance, body adiposity, and regulator of adipocyte metabolism. GPR-39, the obestatin receptor, is lower in obese type 2 diabetes mellitus patients [18]. The expression of mRNA of GPR-39 has a negative relationship with fasting glucose concentration but has a positive relationship with adiponectin mRNA expression, suggesting the involvement of obestatin in glucose homeostasis. An increased level of obestatin is a defense against plasma hyperglycemia. Type 1 and 2 diabetes are characterized by a decrease in beta cell mass induced by apoptosis; thus, the major target is to generate beta cells and promote their survival. Obestatin may be a perfect alternative as it promotes survival and decreases apoptosis of pancreatic beta cells and islet cells. In islet cells, it increases the expression and phosphorylation of insulin receptor substrate and mRNA, which is the main regulator of glucose homeostasis and survival of beta cells [19].

Obestatin in atherosclerosis

It is also called atherosclerotic cardiovascular disease. The build-up of fats and cholesterol in and on the arterial walls causes obstruction of blood flow. The plaque formed may rupture and cause occlusion of the arterial walls [20]. When inflammation of low grade occurs, it is correlated with obesity and is the initial step for progression to atherosclerosis. Obestatin reduces the expression of vascular cell adhesion molecules-1 (VCAM) in endothelial cells and raises the low-density lipoprotein (LDL) level, which binds to macrophages. Decreasing the expression of such adhesion molecules like VCAM-1 proves the anti-atherogenic action of obestatin. In contrast, the rise in LDL uptake by macrophages ultimately leads to the accumulation of lipids and the formation of foam cells, which in turn leads to fatty streak formation in the walls of arteries contributing to proatherogenic properties [21]. There are higher levels of obestatin and ghrelin in individuals with ischemic heart disease compared to normal individuals, suggesting that these peptides have a role in regulating energy metabolism. In addition to these, obestatin also has a role in regulating blood pressure. A positive relationship exists between its level and mean arterial pressure in non-pregnant and pregnant women with pregnancy-induced hypertension [22].

Blood pressure regulation

Plasma obestatin negatively correlates with systolic blood pressure in patients resistant to insulin. There was decreased ghrelin and ghrelin/obestatin ratio in patients with mild to moderate untreated hypertension. In patients with pulmonary arterial hypertension, the obestatin level increases, whereas the ghrelin/obestatin ratio decreases [23,24]. In pregnant females with hypertension, obestatin has a positive relation with hypertension and a negative relation with the mean arterial blood pressure. There were increased levels of obestatin when compared with normotensive people. The obestatin level is also affected by factors like feeding state and diurnal variations [25,26].

The action of obestatin in the heart

Obestatin exerts direct and indirect action in the heart. After binding to GPR-39 on HL-1 cardiac cells, there was no effect on cell viability, cell cycle, fatty acid, or glucose uptake. It reduces the size of infarct and contractile dysfunction in the heart of rats subjected to ischemia-reperfusion and, thus, protects the heart against cell death via activation of various pathways like P13K, PRC-delta, and ERK1/2 pathways. There is a presence of high-affinity obestatin binding sites, which are present in the ventricular myocardium and cardiac cells. In addition, it improves the basal papillary muscle contractility and response to beta-adrenergic receptors stimulation in type 1 diabetes mellitus rats but not in non-diabetic control [27,28]. When administered topically, it induces positive ionotropic effects. Although there is no correlation between obestatin levels in plasma and ischemic heart disease, there is an increased plasma obestatin level in chronic heart failure patients. It suppresses vasopressin and dehydration-induced release of vasopressin, the main regulator of fluid and electrolyte balance [29]. Thus, starting with obestatin inhibits beta-adrenergic response with an endothelial-dependent P13K-AKT-NO-cGMP pathway, which is its main effect. Then, it inhibits endothelinergic response and improves vascular function via the nitric oxide pathway. Then, post-ischemic obestatin inhibits ischemia/reperfusion injury via the nitric oxide-protein kinase G (PKG) pathway. Finally, it counteracts the inhibitory effect of ischemia/reperfusion on the phosphorylation of endothelial nitric oxide synthase (eNOS) and expression of PKG-1 [30,31].

Obestatin-induced nitric oxide pathway

Nitric oxide is a vasoactive gas that freely diffuses into cells, having many physiological roles. The leading function of nitric oxide is cardioprotection. The nitric oxide signaling against ischemia/reperfusion injury is targeted on mitochondria for its action. If nitric oxide signaling is disrupted or inhibited, cardioprotection disappears. The signals originate from the sarcolemmal membrane and then travel to the cytoplasm via the enzyme nitric oxide synthase (NOS), which produces nitric oxide soluble guanylate cyclase (sGC) and PKG. Finally, it is transmitted to the mitochondria, where its action occurs [32].

Myocardial infarction is the major cause of death worldwide. It occurs when the coronary artery is occluded by a thrombotic blood clot or lipid accumulation leading to the obstruction of blood flow, which lasts for 20-40 minutes, leading to tissue damage resulting in infarction. This damage can only be stopped by immediate reperfusion. Nitric oxide is a molecule acting on the cardiovascular system as a vasodilator. In patients with high blood pressure, nitric oxide metabolites are higher than their normal level [33]. It is an important regulator of the contractility of the heart in physiological conditions. In the cardiac cells, all three isoenzymes of nitric oxide are seen, which are neuronal nitric oxide synthase (nNOS), inducible nitric oxide synthase (iNOS), and eNOS. The location of nNOS is the sarcoplasmic reticulum, whereas eNOS is the sarcolemma. nNOS and eNOS are enzymes bound to the membrane and regulate cardiac cells under physiological conditions. Inducible iNOS is a soluble enzyme and is less produced in physiological conditions [34].

Obestatin has inhibitory action under physiological conditions and beta-adrenergic stimulation via nitric oxide signaling. This anti-adrenergic action suggests the protective role of obestatin in protecting the heart against the beta-adrenergic activity of stimulation in stress. The nitric oxide pathway enables obestatin to improve vascular function and oppose the effect of endothelin-1 on vasoconstriction. It exerts post-conditioning effects by decreasing the size of the infarct via the nitric oxide pathway, which phosphorylates eNOS. Obestatin acts on vessels and dilates them, counteracting the vasoconstrictor effect of endothelin-1. This dilatory action is due to the stimulation of the NOS and nitric oxide, released by endothelial cells [35].

## Conclusions

Obestatin is a 23 amino acid peptide originating from a peptide preproghrelin forming an alpha helix. It has a short life and gets degraded rapidly. It has various actions on different sites of the body. The role of obestatin is to inhibit food and water intake, which is known to reduce weight gain and motility of the gastrointestinal tract. It promotes cell survival and prevents apoptosis. It also increases beta cell mass, increases adipogenesis, and induces lipid metabolism. Recently, it has been known to be effective in the cardioprotective role. It has a specific role in controlling blood pressure and is beneficial to endothelial function. It has been effective in ischemia-reperfusion injury. Another gut hormone, ghrelin, is derived from the same peptide of obestatin and has opposite effects to that of obestatin. Though obestatin is still a controversial hormone, research on its action is still underway. Future research focuses on producing stable obestatin peptide, which does not degrade in any conditions.
